# The impact of hyperbaric oxygen therapy on late radiation toxicity and quality of life in breast cancer patients

**DOI:** 10.1007/s10549-021-06332-2

**Published:** 2021-07-19

**Authors:** Marilot C. T. Batenburg, Wies Maarse, Femke van der Leij, Inge O. Baas, Onno Boonstra, Nina Lansdorp, Annemiek Doeksen, Desiree H. J. G. van den Bongard, Helena M. Verkooijen

**Affiliations:** 1 Department of Radiation Oncology, University Medical Center Utrecht, Heidelberglaan 100, 3584 CX Utrecht, The Netherlands; 2 Department of Plastic, Reconstructive and Hand Surgery, University Medical Center Utrecht, Utrecht, The Netherlands; 3 Department of Oncology, University Medical Centre Utrecht, Utrecht, The Netherlands; 4 Medical Director, Institute for Hyperbaric Oxygen Therapy, Rotterdam, The Netherlands; 5 DaVinci Kliniek for Hyperbaric Oxygen Therapy, Rotterdam, The Netherlands; 6 Department of Surgery, St. Antonius Ziekenhuis, Nieuwegein, The Netherlands; 7 Department of Radiation Oncology, Amsterdam University Medical Centers, Amsterdam, The Netherlands; 8 Imaging Division, University Medical Center Utrecht, Utrecht University, Utrecht, The Netherlands

**Keywords:** Breast cancer, Radiation toxicity, Hyperbaric oxygen therapy, Quality of life

## Abstract

**Purpose:**

To evaluate symptoms of late radiation toxicity, side effects, and quality of life in breast cancer patients treated with hyperbaric oxygen therapy (HBOT).

**Methods:**

For this cohort study breast cancer patients treated with HBOT in 5 Dutch facilities were eligible for inclusion. Breast cancer patients with late radiation toxicity treated with ≥ 20 HBOT sessions from 2015 to 2019 were included. Breast and arm symptoms, pain, and quality of life were assessed by means of the EORTC QLQ-C30 and -BR23 before, immediately after, and 3 months after HBOT on a scale of 0–100. Determinants associated with persistent breast pain after HBOT were assessed.

**Results:**

1005/1280 patients were included for analysis. Pain scores decreased significantly from 43.4 before HBOT to 29.7 after 3 months (*p* < 0.001). Breast symptoms decreased significantly from 44.6 at baseline to 28.9 at 3 months follow-up (*p* < 0.001) and arm symptoms decreased significantly from 38.2 at baseline to 27.4 at 3 months follow-up (*p* < 0.001). All quality of life domains improved at the end of HBOT and after 3 months follow-up in comparison to baseline scores. Most prevalent side effects of HBOT were myopia (any grade, *n* = 576, 57.3%) and mild barotrauma (*n* = 179, 17.8%). Moderate/severe side effects were reported in 3.2% (*n* = 32) of the patients. Active smoking during HBOT and shorter time (i.e., median 17.5 vs. 22.0 months) since radiotherapy were associated with persistent breast pain after HBOT.

**Conclusion:**

Breast cancer patients with late radiation toxicity reported reduced pain, breast and arm symptoms, and improved quality of life following treatment with HBOT.

**Supplementary Information:**

The online version contains supplementary material available at 10.1007/s10549-021-06332-2.

## Introduction

Around 68% of all women with breast cancer undergo radiotherapy as part of their treatment [1]. Even though radiotherapy techniques have improved over time, it still may—in combination with systemic therapy and surgery—induce late radiation toxicity [2–4]. Late radiation toxicity is characterized by a combination of breast or chest wall pain, breast and/or arm edema, fibrosis, impaired arm movement, telangiectasia, and impaired cosmetic outcome after radiotherapy. Symptoms such as fibrosis and breast pain may continue to increase during at least 10 years after radiotherapy and substantially impair daily functioning and quality of life [5].

Treatment of late radiation toxicity depends on the symptoms and may consist of analgesics, physiotherapy, lymphedema therapy, and in some cases (reconstructive) surgery. Another proposed treatment for late radiation toxicity is hyperbaric oxygen therapy (HBOT). During HBOT, patients inhale 100% oxygen in a hyperbaric chamber with increased air pressure. The combination of oxygen and increased air pressure induces neovascularization and stimulates formation of collagen by fibroblasts [6, 7]. HBOT has been proven a safe and effective treatment for late radiation toxicity in different tumor sites [8–10]. For that reason, HBOT for late radiation toxicity is endorsed by insurers in the Netherlands. However, evidence for the effectivity of HBOT in breast cancer patients with late radiation toxicity is limited [11, 12]. Consequently, in the Netherlands, HBOT is mostly used as a treatment option for late radiation toxicity in breast cancer patients who insufficiently benefited from analgesics, physiotherapy, or lymphedema therapy.

The aim of this cohort study was to evaluate patient-reported late radiation toxicity in breast cancer patients treated with HBOT between 2015 and 2019 in one center providing hyperbaric oxygen therapy in the Netherlands. Secondly, side effects after HBOT, quality of life, and factors associated with effectivity of treatment were assessed.

## Methods

All breast cancer patients with late radiation toxicity referred between January 2015 and December 2019 for HBOT in the Institute for Hyperbaric Oxygen Therapy (IvHG) were eligible for inclusion. The IvHG has five locations in the Netherlands. Patients who provided written consent for the use of their data for research purposes were included. Patients referred to the IvHG who were found to be ineligible for HBOT (e.g., due to comorbidities), patients treated with < 20 HBOT sessions, or patients referred for re-treatment with HBOT were excluded. Also, patients with osteoradionecrosis and patients treated with HBOT prior to surgery were excluded, as they were treated with a different number of HBOT treatment sessions. Prior to HBOT, a physician confirmed late radiation toxicity and determined if the breast or chest wall symptoms (i.e., a combination of breast or chest wall pain, breast and/or arm edema, fibrosis, impaired arm movement, telangiectasia, and impaired cosmetic outcome) were likely to be the result of radiotherapy. After data collection, the complete dataset was anonymized and transferred to the division of Imaging and Oncology of the UMCU to ensure independent analysis. Data analysis was performed by independent researchers of the UMCU. Staff of the IvHG had no role in study design or decision to file the manuscript for publication. The institutional review board of the University Medical Center Utrecht (UMCU) approved this study.

### Hyperbaric oxygen therapy

Standard HBOT consisted of 40 treatment sessions (1 session/day, 5 days/week) at 2.5 atmospheres absolute (ATA), with a duration of 115 min per session (10 min compression, 4 times 20 min 100% oxygen with breaks of 5 min, and 10 min decompression) [13]. HBOT is administered in a high-pressure chamber. After reaching the desired treatment pressure (2.5 ATA), the patient starts breathing 100% oxygen by a closed built-in breathing system (either a hood or a mask). For safety reasons, the chamber is only filled with air under pressure and the patient always breathes oxygen by a closed system. Patients may receive more or less treatment sessions. For example, treatment effect is evaluated with the HBO physician after 30 treatment sessions. If no treatment effect was seen after 30 sessions, patients could stop HBO treatment after 30 sessions. Also, patients may receive more or less than 40 HBO sessions for other reasons related to HBO (i.e., side effects, sufficient results prior to 40 sessions) or not related to HBO (i.e., planned vacation, medical problems not related to HBO, personal circumstances). Therefore, reasons for treatment sessions other than 40 were recorded. At 3 months after the last HBO session, patients were contacted by phone and received the European Organization for Research and Treatment of Cancer Quality of Life Questionnaire (EORTC QLQ).

### Data collection

Patient, treatment, and tumor characteristics, HBO treatment details, and side effects were extracted from the individual patient records. Patient-reported outcome measures were collected as part of routine clinical care. All data were entered into a database by a research nurse. In accordance with a data collection protocol designed by the UMCU research team, data from the patient files were entered into a standardized case report form. Quality of data extraction was regularly monitored by comparing CRFs with the source documents (around 32 cases, 3%).

### Outcome measurements

#### Patient-reported outcome measurements

Breast/chest wall and arm symptoms, pain, and quality of life were collected as part of standard care using the EORTC QLQ. The EORTC QLQ comprises 30 quality of life and functioning items (C30) as well as 23 breast-specific items (BR23) [14]. All items were scored on a 4-point Likert scale. Total scores (0–100) for subscales of the EORTC questionnaires were calculated using the EORTC scoring manual. For functional scales, a higher score indicated a better outcome. For symptom scales, a higher score indicated more symptoms. Breast symptoms were evaluated using four questions on pain, swelling, sensitivity, and skin problems in the affected breast or chest wall (BR23). The arm symptom scale is based on 3 items: pain and swelling in arm or shoulder and difficulty to move the arm up or sideways. The EORTC QLQ questionnaires were used as part of standard treatment. Patients received questionnaires at predefined time-points, i.e., prior to treatment (baseline), after the last HBO session (2 months after baseline), and at 3 months after the last HBO session (5 months after baseline).

#### Cohort outcomes and side effects

Side effects of HBOT were evaluated by the HBO physician during follow-up visits (i.e., after 15, 30, and 40 sessions and by telephone at 3 months after the end of HBOT). Side effects after HBOT may include barotrauma, hypoglycemia, myopia, fatigue, cataract, sinus squeeze, (acute or chronic) oxygen toxicity, cardiac decompensation/heart failure, decompression disease, or pneumothorax. Otoscopy was only performed in case of ear pain or repetitive trouble in equalizing middle ear pressure. Then, barotrauma was classified according to the 6-point MacFie classification (also known as modified TEED classification): no abnormalities with otoscopy (grade 0), increased vessel visibility around the eardrum (without/with minor/with major bleeding, grade 1–3), blood in middle ear (grade 4), or eardrum perforation (grade 5) [15–17]. All side effects were standardly evaluated during visits with the HBOT physician. However, no grading system was available for other side effects than barotrauma. For this study, fatigue was evaluated using the EORTC QLQ-C30 fatigue subscale. A fatigue score ≥ 71 was considered clinically relevant, based on the Thresholds for Clinical Importance of Giesinger et al. [18]. Newly developed (clinically relevant) fatigue during HBOT or at follow-up was considered to be a side effect of the HBOT. Barotrauma grade 0–2, hypoglycemia, myopia, and fatigue were classified as mild side effects, as they are transient in nature [19]. Moderate or severe side effects were cataract, barotrauma grade 3–5, sinus squeeze, (acute or chronic) oxygen toxicity, cardiac decompensation/heart failure, decompression disease, or pneumothorax.

### Statistics

Patient characteristics, breast cancer treatment, HBO treatment characteristics, and side effects were described using frequencies and proportions for categorical data and for continuous data means with standard deviation for normally distributed data and medians with interquartile ranges (IQR) were used for skewed data.

Paired T tests or Wilcoxon rank test—depending on distribution—were used to compare pain, breast symptoms, and arm symptoms between baseline (T0) and T1 (end treatment) and between T0 and T2 (follow-up), respectively. Analysis was performed using all available questionnaires. For sensitivity analysis, complete case analysis was performed. To evaluate the association between patient and treatment characteristics and persistence of breast pain after HBOT, the EORTC QLQ-BR23 item on breast pain was used (item 50, “Have you had any pain in the area of your affected breast?”). Breast pain was dichotomized into moderate/severe pain and no/mild pain. Patients with persistent moderate/severe breast pain after HBOT were categorized as unsuccessful therapy (no pain response). Descriptive statistics were used to evaluate characteristics associated with adequate treatment effect, i.e., mild or no pain at follow-up. Statistical Package for Social Sciences (SPSS) software version 25 was used for analysis. A *p*-value < 0.05 was considered significant.

## Results

**Fig. 1:**
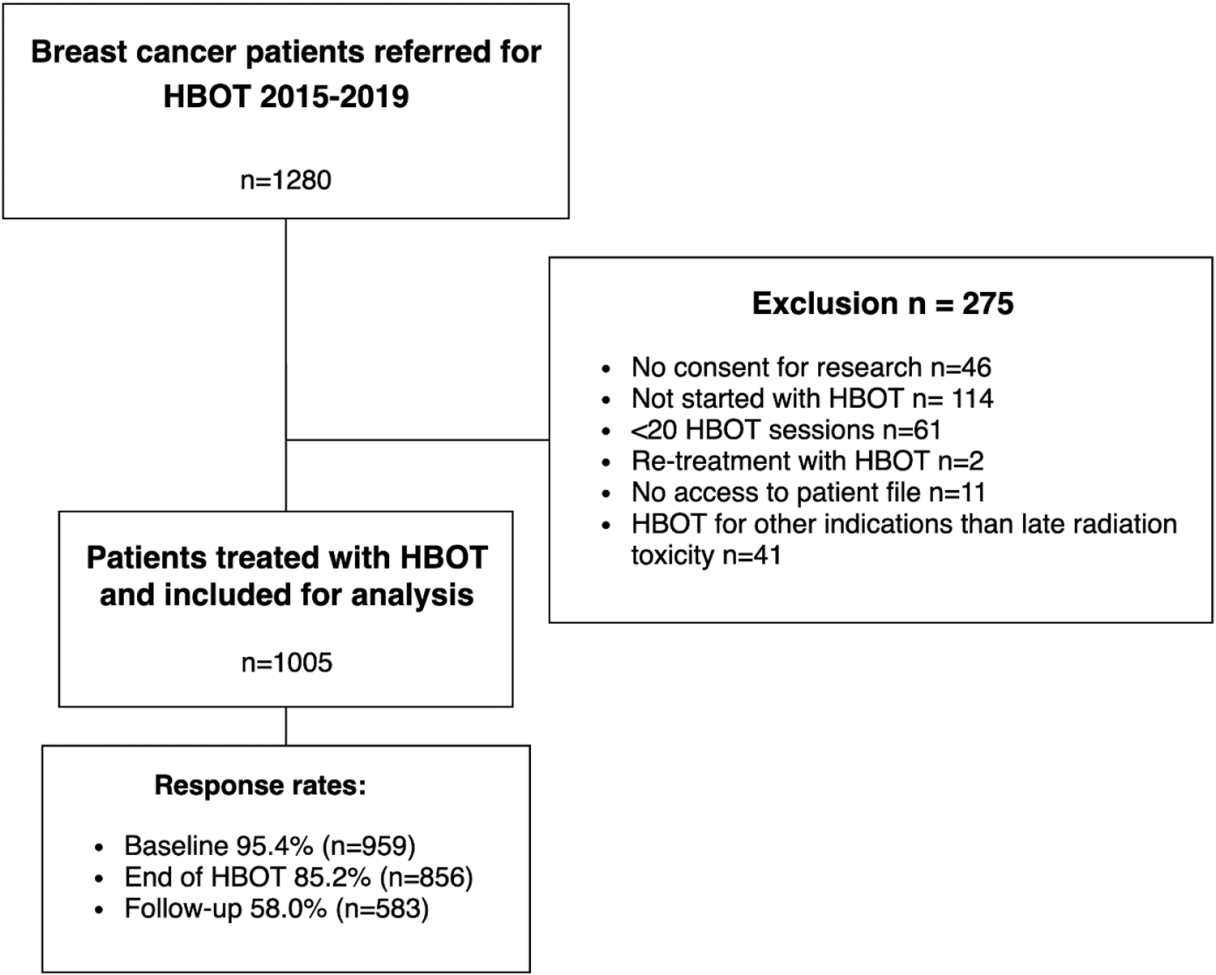
Flowchart of patients included for analysis after in- and ex-clusion criteria

Between January 2015 and December 2019, 1280 breast cancer patients were referred for HBOT. Of those, 1005 (78.5%) patients were included for analysis (Fig. 1). The most common reasons for exclusion were ineligibility for HBOT (*n* = 114), treatment with < 20 HBOT sessions (*n* = 61), and no consent for the use of data for research (*n* = 46). The response rate to the EORTC questionnaire was 95% at baseline, 85% at the end of treatment, and 58% after 3 months follow-up. The majority of patients were female (*n* = 1002, 99.7%) (Table 1). The mean age was 57.9 years and most patients were treated with breast-conserving surgery (*n* = 731, 73%). The most common radiotherapy fractionation schedule was 15–19 fractions without boost (*n* = 231, 23.0%) or 21–24 fractions with boost (*n* = 176, 17.5%). In total, 336 (33.4%) patients received local radiotherapy and 264 (26.3%) patients received locoregional radiotherapy (i.e., radiation therapy on periclavicular and/or axillary lymph nodes). During HBOT, 13% (*n* = 134) of the patients were active smokers and 41% (*n* = 413) were former smokers. The time since radiotherapy ranged from 1 to 582 months (median 22 months). Patients who responded to all questionnaires were, on average, older (mean age 59.0 vs. 56.8) and had a longer time since radiotherapy (median 48 months vs. 37 months) than non-responders (Supplementary material Table 1).

**Table 1:** Baseline characteristics

	*n* = 1005
Age [mean (SD)]	57.9 (9.7)
Female gender	1002 (99.7)
Pathological tumor stage^a^	
0	4 (0.4)
In situ	34 (3.4)
1	456 (45.4)
2	246 (24.5)
3	56 (5.6)
4	18 (1.8)
Unknown	191 (19)
Type of surgery	
Breast-conserving surgery	731 (72.7)
Mastectomy without breast reconstruction	180 (17.9)
Autologous breast reconstruction	36 (3.6)
Implant breast reconstruction	29 (2.9)
Breast reconstruction, unknown type	17 (1.7)
Unknown	12 (1.2)
Axillary surgery^a^	
Axillary lymph node dissection	257 (25.5)
Sentinel Node Procedure	569 (56.6)
Other	10 (1.0)
No axillary treatment/unknown	169 (16.8)
Systemic treatment	
Chemotherapy alone	161 (16.0)
Hormonal therapy alone	106 (10.5)
Both chemotherapy and hormonal therapy	464 (46.2)
No adjuvant treatment	241 (24.0)
Unknown	33 (3.3)
Smoking	
Never	455 (45.3)
Current smoker	134 (13.3)
Previous smoker	413 (41.1)
Unknown	3 (0.3)
Diabetes Mellitus	
Yes	83 (8.3)
No	922 (91.7)
Body Mass Index (median (IQR))^b^	27.4 (7.1)
Unknown	228 (25.3)
Type of radiation therapy	
Local	336 (33.4)
Locoregional	264 (26.3)
Unknown	405 (40.3)
Radiotherapy boost^c^	
Yes	372 (39.4)
No	396 (37.0)
Unknown	237 (23.6)
Radiotherapy fractionation^d^	
6–12 fractions	15 (1.5)
15–19 fractions	231 (23.0)
21–24 fractions, with boost	176 (17.5)
20–25 fractions, no boost	122 (12.1)
> 26 fractions	88 (8.8)
Unknown	373 (37.1)
Previous radiotherapy breast/chest wall^a^	
Yes	51 (5.1)
No	699 (69.6)
Unknown	255 (25.4)
Months since radiotherapy [median (IQR)]	22 (35)

Numbers are shown as n(%) unless stated otherwise. Continuous outcomes are shown as mean (SD) when normally distributed and median(IQR) otherwise

*SD* standard deviation, *IQR* interquartile range

^a^Total other than 100% due to rounding

^b^Calculated as weight/height^2^

^c^An additional radiotherapy boost on the tumor bed or axillary/lymph node boost

^d^Dose per fraction was unknown

The number of HBO sessions ranged from 20 to 60 (median 40); 73.1% (*n* = 735) of the patients received 40 HBO sessions (Table 2). Reasons for undergoing less HBOT than planned were personal circumstances (*n* = 53), sufficient results (*n* = 31), or medical problems not related to HBOT (*n* = 29). There were 32 patients who stopped HBOT early due to no or insufficient results and 17 patients who stopped due to complications of HBOT. In total, 30 patients received > 40 HBOT sessions, mostly due to disruption of treatment sessions (*n* = 13). The most common side effects of HBOT were (transient) myopia (*n* = 576, 57%) and mild barotrauma (*n* = 179, 18%) (Table 2). Moderate/severe side effects were reported by 32 patients: oxygen toxicity (*n* = 4, 0.4%), barotrauma grade 3–4 (*n* = 26, 2.6%), sinus squeeze (*n* = 1, 0.1%), and cataract (*n* = 1, 0.1%).

**Table 2:** Number of hyperbaric oxygen treatment sessions, reasons for treatment sessions < 40, and side effect of hyperbaric oxygen therapy

Number of HBO sessions	*n* = 1005
HBO sessions [median(range)]	40 (20–60)
< 40 sessions (n (%))	240 (23.9)
40 sessions [n (%)]	735 (73.1)
> 40 sessions [n (%)]	30 (3.0)

Reasons for treatment sessions < 40	*n* (%)
Sufficient results	31 (13)
No/insufficient results	32 (13)
Complications of HBOT	17 (7)
Private circumstances	53 (22)
Medical problems not related to HBOT	29 (12)
Unclear	78 (33)
Total	240 (100)

Side effects of HBOT	
Number of patients with side effects [n (%)]	697 (69.4)
Number of side effects	882
Mild (transient) side effects	*n* (%)
Barotrauma grade 0–2^a^	179 (17.8)
Hypoglycemia	2 (0.2)
Myopia	576 (57.3)
Fatigue (newly developed)	52 (5.2)
Complication, unclear	41 (4.1)
Moderate/severe side effects	*n* (%)
Cataract^b^	1 (0.1)
Barotrauma grade 3–4^a^	26 (2.6)
Barotrauma sinus squeeze	1 (0.1)
Oxygen toxicity	4 (0.4)

No cases: chronic oxygen toxicity, cardiac decompensation/heart failure, decompression disease, hypoxia, deceased, pneumothorax. Fatigue was calculated as number of patients with newly developed fatigue during HBOT [i.e., fatigue scores higher than 40 (18)]

*HBOT* hyperbaric oxygen therapy

^a^In accordance with the Macfie classification

^b^Cataract may be therapy induced or pre-existent

**Fig. 2:**
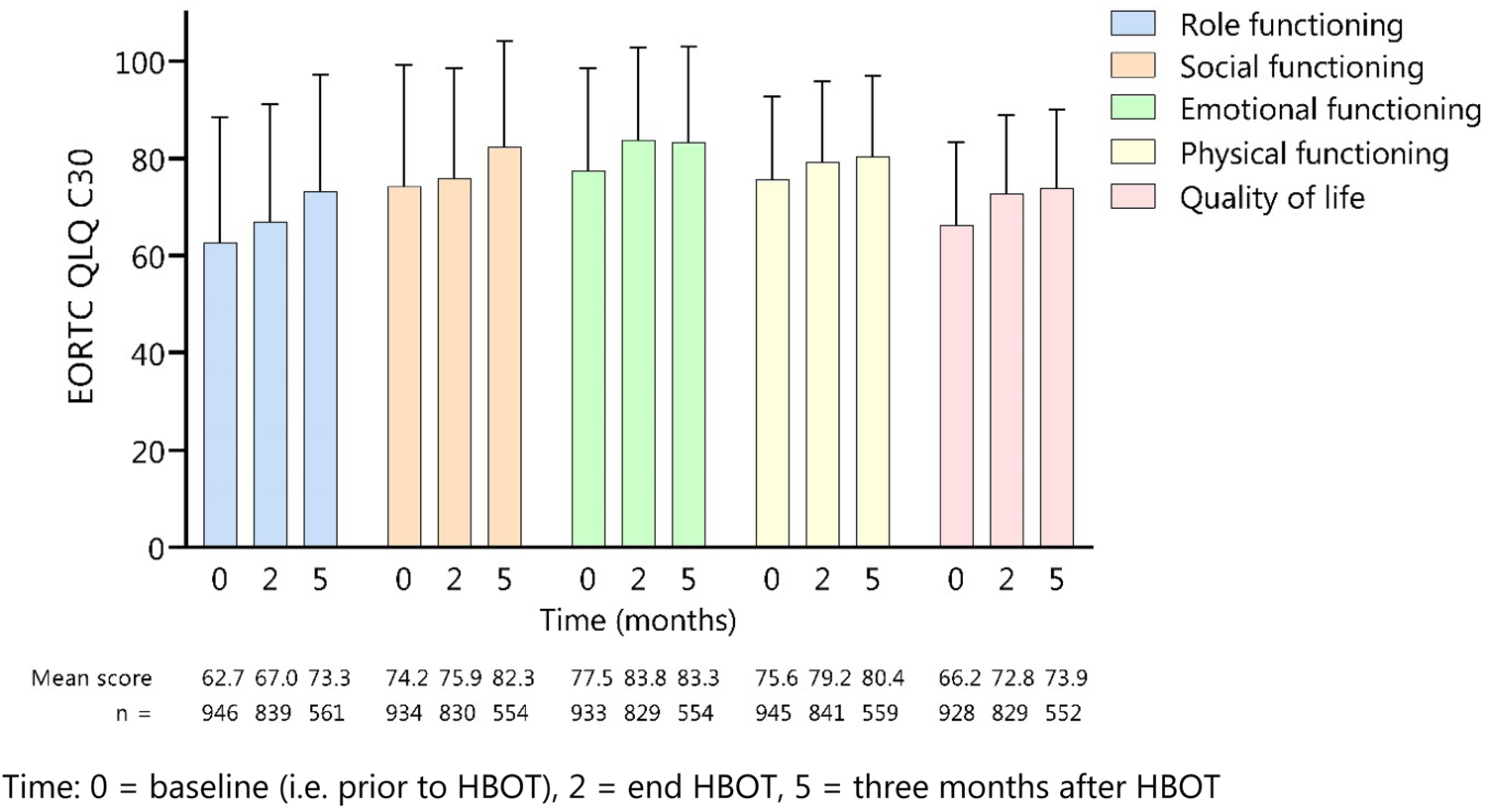
The effect of hyperbaric oxygen therapy on pain, breast symptoms, and arm symptoms. A higher score indicates more symptoms. *Significant difference (*p* < 0.05) tested with Wilcoxon rank test. Time: 0 = baseline (i.e., prior to HBOT), 2 = end of HBOT, 5 = 3 months after HBOT

**Fig. 3:**
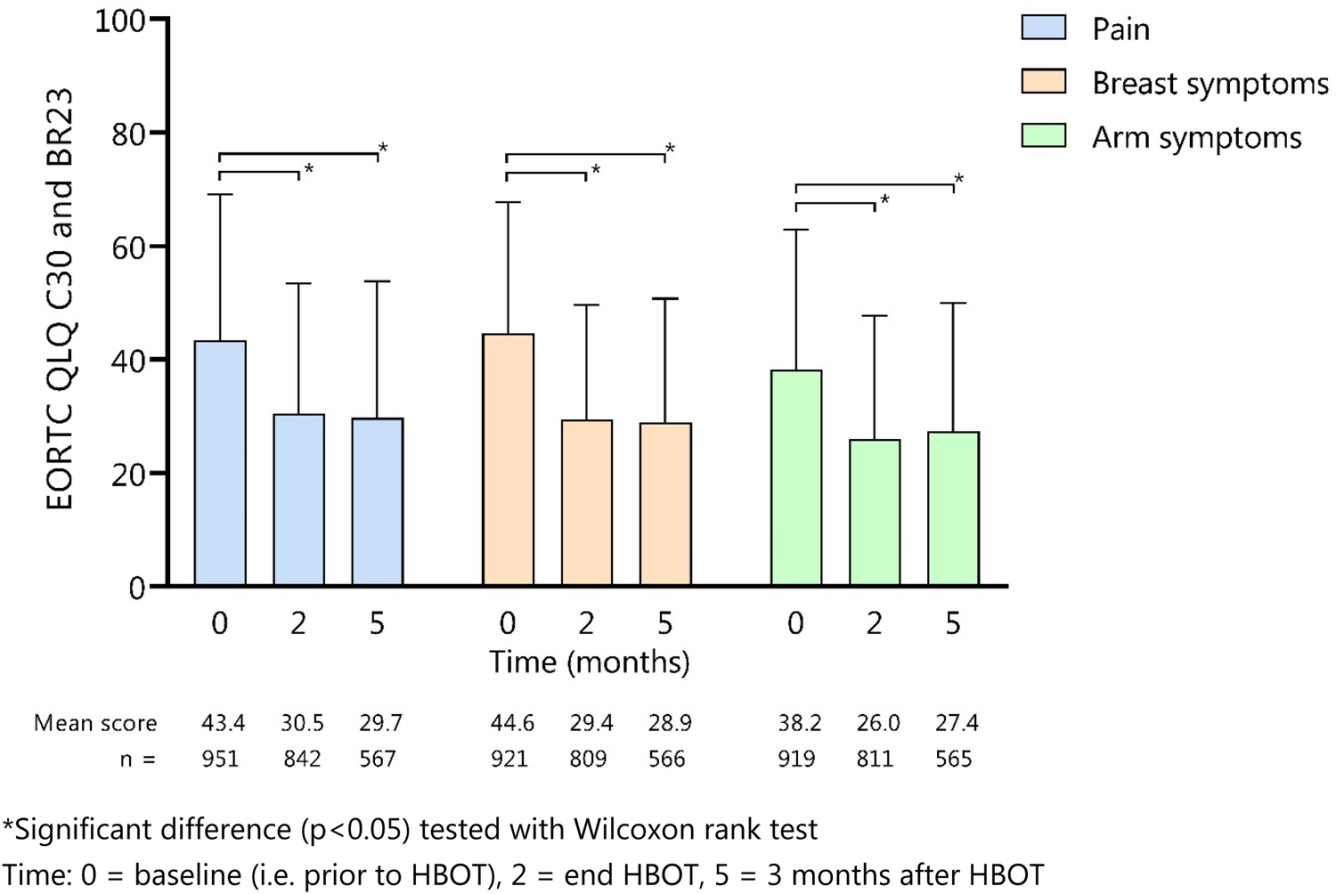
The effect of hyperbaric oxygen therapy on quality of life scores and role, emotional, social, and physical functioning using the EORTC QLQ-C30 questionnaire. A higher score indicates a better quality of life. Time: 0 = baseline (i.e., prior to HBOT), 2 = end of HBOT, 5 = three months after HBOT

Pain scores decreased significantly from 43.4 prior to HBOT to 30.5 at the end of HBOT (*p* < 0.001) to 29.7 at 3 months follow-up (*p* < 0.001) (Fig. 2). Also, a significant reduction in breast symptom scores at the end of HBOT (29.4) and 3 months follow-up (28.9) was seen in comparison to baseline score (44.6) (*p* < 0.001). Arm symptom scores reduced significantly (*p* < 0.001) from 38.2 to 26.0 at the end of treatment and 27.4 after 3 months follow-up. Repeating the analysis in the subgroup of 352 patients who completed questionnaires at all timepoints did not change the results (Supplementary table 2). Role functioning scores improved from 62.7 at baseline to 67.0 immediately after HBO and 73.2 after 3 months follow-up (Fig. 3). Social functioning scores improved from 74.2 prior to treatment to 75.9 after treatment and further to 82.3 after 3 months follow-up. Also, emotional functioning, physical functioning, and quality of life scores increased over time.

EORTC breast pain scores were available at baseline and at the end of HBOT for 749 patients. In total, 61.5% (*n* = 461/749) of the patients reported breast pain grade 3–4 prior to treatment and 30.0% (*n* = 225/749) reported breast pain grade 3–4 after HBOT. Of the patients with pain grade 3–4 at baseline, 271 patients (58.8%) had grade 1–2 pain at end of treatment and 190 patients still had pain grade 3–4 (i.e., treatment failures) after HBOT (Table 3). Factors associated with treatment success were smoking and time since radiotherapy. Of the patients who smoked during HBOT, 45% (*n* = 29/64) had good response (i.e., no/mild pain after HBOT), 61% (*n* = 121/199) of the never smokers and 61% of the former smokers (*n* = 120/198) had good response to HBOT. The median time since radiotherapy was 22 months in the group with good response to HBOT and 17.5 months in the group with persistent pain after HBOT.

**Table 3:** Characteristics of patients with and without persistent breast or chest wall pain after hyperbaric oxygen therapy

	Pain response (*n* = 271)	No pain response (*n* = 190)
Age [mean (SD)]	57.9 (9.7)	57.4 (8.9)
Type of surgery		
Breast-conserving surgery	206 (57)	153 (43)
Mastectomy without breast reconstruction	49 (66)	25 (34)
Mastectomy followed by breast reconstruction^a^	13 (57)	10 (44)
Unknown	3 (60)	2 (40)
Systemic treatment		
Chemotherapy alone	40 (56)	31 (44)
Hormonal therapy alone	38 (62)	23 (38)
Both chemotherapy and hormonal therapy	115 (60)	76 (40)
No (neo)adjuvant treatment	68 (54)	58 (46)
Smoking		
Never	121 (61)	77 (39)
Current smoker	29 (45)	35 (54)
Previous smoker	120 (61)	78 (39)
Unknown	1 (100)	0 (0)
Diabetes Mellitus		
Yes	18 (55)	15 (46)
No	253 (59)	175 (41)
Body Mass Index (median IQR)^b^	27.9 (7.1)	26.6 (7.2)
Radiotherapy boost		
Yes	98 (57)	75 (43)
No	111 (63)	64 (37)
Unknown	62 (55)	51 (45)
Months since radiotherapy [median(IQR)]	22 (34)	17.5 (30)

Numbers are shown as n (%) unless stated otherwise. Continuous outcomes are shown as mean (SD) when normally distributed and median (IQR) otherwise

Patients with breast pain grade 3–4 (EORTC QLQ 50) at baseline were selected. Patients without breast pain were defined as breast pain grade 1–2 at end of HBOT. Patients with breast pain were defined as patients with grade 3–4 breast pain at the end of HBOT

*SD* standard deviation, *IQR* interquartile range

^a^Total other than 100% due to rounding

^b^Calculated as weight/height^2^

## Discussion

In this large cohort study of breast cancer patients with late radiation toxicity, a reduction of pain, breast and arm symptoms, and an improvement in patient-reported outcomes (i.e., quality of life and social, role, emotional, and physical functioning) following treatment with HBOT was seen. The majority of the patients in this study experienced some side effects of HBOT. The most common side effects were (transient) myopia and mild barotrauma. Myopia and mild barotrauma are transient side effects and disappear mostly in the first three months after HBOT. This study confirmed that HBOT is a safe treatment, as severe side effects were seen in 3.6% of all patients and mostly concerned barotrauma’s.

Two previous studies evaluated the effect of HBOT for breast cancer patients with late radiation toxicity. In the prospective cohort study by Carl et al., outcomes of 32 breast cancer patients treated with HBOT were compared with 12 control patients who refused HBOT [12]. Late radiation toxicity was evaluated using the LENT-SOMA scores on a 4-point Likert scale. Similar to our study, a significant reduction in pain was seen after HBOT. Eleven months after treatment, median pain scores for the HBOT group decreased from 3 (range 1–4) prior to HBOT to 0 (range 0–2). The median pain score in the observational group remained stable at grade 3 over time. Like us, Carl et al. reported a significant reduction of edema after HBOT. This reduction of edema was not seen in the control group. In contrast to our study, no effect on physician-reported fibrosis was reported by Carl. et al. In the study by Carl et al., the median fibrosis score was already 0 in both groups prior to the study; so, no effect of HBOT on fibrosis could be seen.

In the prospective study by Teguh et al., 57 patients with late radiation toxicity received on average 47 HBO sessions on 2.4 ATA [11]. Late radiation toxicity was evaluated by means of the EORTC QLQ-C30 and -BR23. Moderate/severe breast pain was seen in 66.7% of the patients prior to HBOT, which is similar to 61.5% in our study. At the end of HBOT, 14.5% of the patients reported moderate/severe pain. This proportion was 30.0% in our study. In the study from Teguh et al., 51% of the patients received chemotherapy and 6/57 (11%) of the patients had no surgery in contrast to, respectively, 72% and at most 1.2% in our population. Consequently, there might be more fibrosis in our population and treatment with HBOT could therefore have been less effective. Proportions of moderate/severe swelling of breast and arm and problems with moving the arm prior to HBOT and after HBOT in the study of Teguh et al. were comparable to our study.

In our study, pain response was defined as a decrease in pain from grade 3–4 to 1–2 after HBOT. The proportion of patients that still experienced pain after HBOT was higher in the group of patients that actively smoked in comparison to patients who were never or former smokers. HBOT induces neo-vascularization and smoking might damage these newly developed vessels [6]. Consequently, patients who actively smoke during treatment might have less effect of the treatment and experience persisting breast pain after HBOT. In addition, the interval between radiotherapy and HBOT was slightly larger (i.e., difference of 5 months) for patients with breast pain response than for patients with persistent pain after HBOT. A possible explanation is that when radiotherapy is longer ago, it could be more straightforward to differentiate late radiation toxicity from side effects of other breast cancer treatments. As HBOT is specifically targeted for late radiation toxicity, better selection of patients eligible for HBOT may lead to better treatment results. Also, patients who suffered longer from breast pain may report a larger difference in breast pain as they are more relieved than patients who suffered breast pain shortly.

Our study suffers from several limitations: first, clinical outcome data were collected retrospectively, which may have introduced some room for information bias. For example, there may be an underestimation of side effects of HBOT as, theoretically, not all physicians consequently reported side effects in the patient records. To ensure data quality, independent monitoring of extracted data was performed. While monitoring, no discrepancies in extracted data and source date were seen. Second, despite a very high response rate at baseline and at the end of treatment, the response rate at 3 months after the end of treatment was suboptimal (58%). This is partly due to the fact that not all patients were contacted at 3 months after HBOT. Also, the response rate depends on the response of the patients to the EORTC QLQ. In case the response was selective, this may have over- or under-estimated the impact of HBOT on PROs. Some patient characteristics differed between non-responders and responders, as non-responders were on average older and received radiotherapy longer ago. Also, the reason for non-response is unknown. Therefore, the effect of HBOT could have been different for non-responders than responders. Third, no long-term follow-up was available for this study and no control group was included. Potentially, symptoms and quality of life could also have improved over time (i.e., regressed to the mean) without treatment of HBOT [20, 21]. As there was no control group, no distinction could be made between regression to the mean and the effect of HBOT. Therefore, the study results need to be confirmed in a randomized controlled trial in order to compare HBOT to a control group. For that reason, we are currently conducting a randomized controlled trial following the Trials within Cohorts design in our institute (NCT04193722) [22]. In this trial, the effect of HBOT on late radiation toxicity is compared to usual care in breast cancer patients.

In conclusion, this large study of consecutive breast cancer patients with late radiation toxicity shows a beneficial effect of HBOT on patient-reported symptoms and quality of life and functioning until at least three months after HBOT. Also, it confirms that hyperbaric oxygen therapy is safe, as severe side effects were limited. The most common side effects were (reversible) myopia and mild barotrauma. Due to the non-comparative design of the study, these results need to be confirmed in a randomized controlled trial.

## Supplementary Information

Below is the link to the electronic supplementary material.

Supplementary file1 (DOCX 18 kb)

## Data Availability

Data are available upon request.

## Code availability

Data were analyzed anonymously. Code was available by the research nurse of the IvHG.
