# Supplementary material for: The impact of hyperbaric oxygen therapy on late radiation toxicity and quality of life in breast cancer patients
Source: Breast Cancer Res Treat. 2021 Jul 19;189(2):425–33. doi: 10.1007/s10549-021-06332-2 (PMC8357739; doi:10.1007/s10549-021-06332-2)
Supplement: Supplementary file 1 — Supplementary file1 (DOCX 18 kb) [file 10549_2021_6332_MOESM1_ESM.docx]

**Supplementary table 1.** Characteristics of responders vs. non-responders to questionnaires.

|  | Questionnaire responders (n=526) | Non-responders to questionnaires (n=479) |
| --- | --- | --- |
| **Age (mean (SD))** | 59.0 (9.6) | 56.8 (9.7) |
| **Type of surgery^a^** |  |  |
| Breast conserving surgery | 379 (72) | 352 (74) |
| Mastectomy without breast reconstruction | 104 (20) | 76 (16) |
| Mastectomy followed by breast reconstruction | 40 (8) | 42 (9) |
| Unknown | 3 (1) | 9 (2) |
| **Axillary surgery^a^** |  |  |
| Axillary lymph node dissection | 141 (27) | 116 (24) |
| Sentinel Node Procedure | 293 (56) | 276 (58) |
| Other | 5 (1) | 5 (1) |
| No/unknown | 87 (17) | 82 (17) |
| **Systemic treatment^a^** |  |  |
| Chemotherapy alone | 83 (16) | 78 (16) |
| Hormonal therapy alone | 64 (12) | 42 (9) |
| Both chemotherapy and hormonal therapy | 247 (47) | 217 (45) |
| No (neo)adjuvant treatment | 115 (22) | 126 (26) |
| Unknown | 17 (3) | 16 (3) |
| **Smoking** |  |  |
| Never | 239 (45) | 216 (45) |
| Current smoker | 65 (12) | 69 (14) |
| Previous smoker | 221 (42) | 192 (40) |
| Unknown | 1 (0.2) | 2 (0.4) |
| **Diabetes Mellitus** |  |  |
| Yes | 41 (8) | 42 (9) |
| No | 485 (92) | 437 (91) |
| **Body Mass Index (median (IQR))^b^** | 28.0 (6.8) | 28.3 (7.8) |
| **Radiotherapy fractionation^a^** |  |  |
| 6-12 fractions | 8 (2) | 7 (2) |
| 15-19 fractions | 120 (23) | 111 (23) |
| 21-24 fractions, including boost | 91 (17) | 85 (18) |
| 20-25 fractions, no boost | 63 (12) | 59 (12) |
| >26 fractions | 47 (9) | 41 (9) |
| Unknown | 197 (38) | 176 (37) |
| **Months since radiotherapy (median(IQR))** | 48.0 (41) | 36.9 (31) |

Numbers are shown as n(%) unless stated otherwise. Continuous outcomes are shown as mean(SD) when normally distributed and median(IQR) otherwise. Responders was defined as patients who filled in all questionnaires (i.e. baseline, at end of treatment and at 3 months follow-up after HBOT).

^a^ Total percentage other than 100% due to rounding. ^b^ Calculated as weight/height^2^

Abbreviations: IQR interquartile range, SD standard deviation

**Supplementary table 2.** The effect of hyperbaric oxygen therapy on pain, breast symptoms and arm symptoms using all available cases vs. complete cases

|  |  | **Pain** | | | **Breast symptoms** | | | **Arm symptoms** | | |
| --- | --- | --- | --- | --- | --- | --- | --- | --- | --- | --- |
|  |  | 0 | 2 | 5 | 0 | 2 | 5 | 0 | 2 | 5 |
| All available cases | Mean | 43.4 | 30.5 | 29.7 | 44.6 | 29.4 | 28.9 | 38.2 | 26.0 | 27.4 |
|  | n | 951 | 842 | 567 | 921 | 809 | 566 | 919 | 811 | 565 |
|  | p-value | Ref. | <0.001 | <0.001 | Ref. | <0.001 | <0.001 | Ref. | <0.001 | <0.001 |
| Complete case analysis | Mean | 42.4 | 30.6 | 29.2 | 44.5 | 30.4 | 28.8 | 37.8 | 26.7 | 27.4 |
|  | n | 352 | 352 | 352 | 352 | 352 | 352 | 352 | 352 | 352 |
|  | p-value | Ref. | <0.001 | <0.001 | Ref. | <0.001 | <0.001 | Ref. | <0.001 | <0.001 |

Time: 0 = baseline (i.e. prior to HBOT), 2 = end HBOT, 5 = three months after HBOT

Ref. = reference category

A complete case was defined as a patient who filled in the EORTC questionnaires on all time points. Differences between T0 and resp. T1 and T2 were calculated using a Wilcoxon rank test.

Pain, breast symptoms and arm symptoms were calculated by means of the EORTC QLQ C30 and BR23. Scores ranged from 0-100. A higher score indicated more symptoms.
